# Heterologous expression, biochemical characterization, and overproduction of alkaline α-amylase from *Bacillus alcalophilus *in *Bacillus subtilis*

**DOI:** 10.1186/1475-2859-10-77

**Published:** 2011-10-07

**Authors:** Haiquan Yang, Long Liu, Jianghua Li, Guocheng Du, Jian Chen

**Affiliations:** 1 Key Laboratory of Industrial Biotechnology, Ministry of Education, Jiangnan University, Wuxi 214122, China; 2 School of Biotechnology, Jiangnan University, Wuxi 214122, China; 3 State Key Laboratory of Food Science and Technology, Jiangnan University, Wuxi 214122, China

## Abstract

**Background:**

Alkaline α-amylases have potential applications for hydrolyzing starch under high pH conditions in the starch and textile industries and as ingredients in detergents for automatic dishwashers and laundries. While the alkaline α-amylase gains increased industrial interest, the yield of alkaline α-amylases from wild-type microbes is low, and the combination of genetic engineering and process optimization is necessary to achieve the overproduction of alkaline α-amylase.

**Results:**

The alkaline α-amylase gene from *Bacillus alcalophilus *JN21 (CCTCC NO. M 2011229) was cloned and expressed in *Bacillus subtilis *strain WB600 with vector pMA5. The recombinant alkaline α-amylase was stable at pH from 7.0 to 11.0 and temperature below 40°C. The optimum pH and temperature of alkaline α-amylase was 9.0 and 50°C, respectively. Using soluble starch as the substrate, the *K*_m _and *V*_max _of alkaline α-amylase were 9.64 g/L and 0.80 g/(L·min), respectively. The effects of medium compositions (starch, peptone, and soybean meal) and temperature on the recombinant production of alkaline α-amylase in *B. subtilis *were investigated. Under the optimal conditions (starch concentration 0.6% (w/v), peptone concentration 1.45% (w/v), soybean meal concentration 1.3% (w/v), and temperature 37°C), the highest yield of alkaline α-amylase reached 415 U/mL. The yield of alkaline α-amylase in a 3-L fermentor reached 441 U/mL, which was 79 times that of native alkaline α-amylase from *B. alcalophilus *JN21.

**Conclusions:**

This is the first report concerning the heterologous expression of alkaline α-amylase in *B. subtilis*, and the obtained results make it feasible to achieve the industrial production of alkaline α-amylase with the recombinant *B. subtilis*.

## Background

α-Amylases (1, 4-α-D-glucan glucanohydrolase; EC 3.2.1.1) are endo-acting enzymes that hydrolyze starch by cleaving α-1, 4-glucosidic linkages [1], and have been widely used in food, textile, and pharmaceutical industries [2-7]. The alkaline α-amylases have high catalytic efficiency and stability at the alkaline pH ranging from 9 to 11 [8-11], and have potential applications for hydrolyzing starch under high pH conditions in the starch and textile industries and as ingredients in detergents for automatic dishwashers and laundries [3-8].

While the alkaline α-amylases have potential applications in textile and detergent industries, relatively few efforts have been made to improve the yield of alkaline α-amylases. The current studies mainly focus on the strain screening, enzyme purification, and properties characterization [3,9,12-19].

The yield of alkaline α-amylase from wild-type microbes, especially from extremophilic microbes, is usually low and hard to be improved, even with process optimization and control [13,14,16]. It is necessary to use genetic engineering to clone and express the alkaline amylase gene in a suitable host for the further improvement of enzyme yield. The alkaline α-amylase from *Bacillus halodurans *MS-2-5 and *Bacillus halodurans *38C-2-1 was expressed in *Escherichia coli *and the highest yield reached 52 U/mL under the optimal conditions [3,7]. Compared with *E. coli*, *Bacillus subtilis *is non-pathogenic and bacteriophage-resistant, and has been extensively used for the overproduction of heterologous proteins [20-28]. Nevertheless, the cloning and expression of alkaline α-amylase gene in *B. subtilis *have not been reported.

In our previous work, a strain capable of producing alkaline α-amylase was isolated and identified as *Bacillus alcalophilus *JN21 (CCTCC NO. M 2011229), and the optimum pH and temperature of the alkaline α-amylase were 9.5 and 55°C, respectively [29]. In this work, *B. subtilis *expression system was constructed to clone and express the alkaline α-amylase from *B. alcalophilus *JN21. The biochemical characterization of the recombinant alkaline α-amylase was also performed. To achieve the over-production of alkaline α-amylase in *B. subtilis*, the key media components (starch, peptone, and soybean meal) and temperature were optimized in shaker flask. The production of alkaline α-amylase by *B. subtilis *was conducted in a 3-L fermentor under the obtained optimal conditions. The results obtained here may be useful for the achievement of industrial alkaline α-amylase production.

## Results and Discussion

### Expression of recombinant alkaline α-amylase in *B. subtilis*

**Figure 1:**
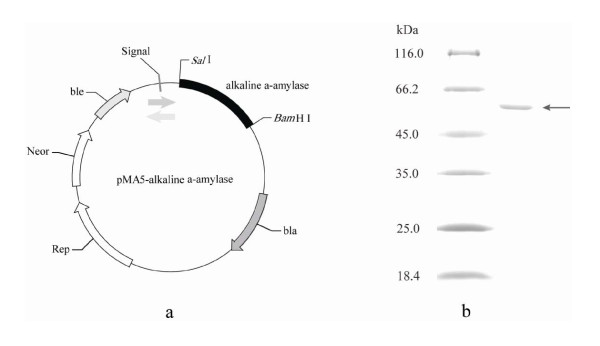
**Map of expression vector and SDS-PAGE analysis**. a: Map of expression vector pMA5-alkaline α-amylase. b: SDS-PAGE analysis of purified alkaline α-amylase. Fragment indicated by arrow: alkaline α-amylase.

DNA fragment coding for mature alkaline α-amylase was obtained from *B. alcalophilus *JN21 by PCR and cloned into the *B. subtilis *expression vector, pMA5 (Figure 1(a)). Nucleotide sequence analysis showed that the gene length was 1671 bp and the encoded protein consisted of 557 amino acids. The target fragment was integrated into *B. subtilis *WB600 strain. The transformants were selected at 37°C on the LB agar plates containing 100 μg/mL Kanamycin and 1% soluble starch. The transformants with transparent rings around colonies were positive. The insertion of alkaline α-amylase gene in the transformants was confirmed by PCR. The *B. subtilis *WB600 transformed with vector pMA5 was used as the control. The colonies were grown in 25 mL of LB medium at 37°C for 36 h in 250 mL shaker flasks. The alkaline α-amylase activity in the culture supernatant was 99 U/mL. The alkaline α-amylase activity in the culture supernatant of control strain was determined under the same culture conditions and the activity was 4 U/mL. The yield of recombinant alkaline α-amylase in the culture media of engineered *B. subtilis *was 18 times that of native alkaline α-amylase from *B. alcalophilus *JN21.

SDS-PAGE analysis showed that there was one major band of protein (approximate 56 kDa) secreted into the culture medium (Figure 1(b)). The recombinant alkaline α-amylase was purified from culture supernatant by the anion exchange (Q-Sepharose HP), and the specific activity of purified enzyme was 168 U/mg.

### Effect of pH and temperature on the activity and stability of alkaline α-amylase

**Figure 2:**
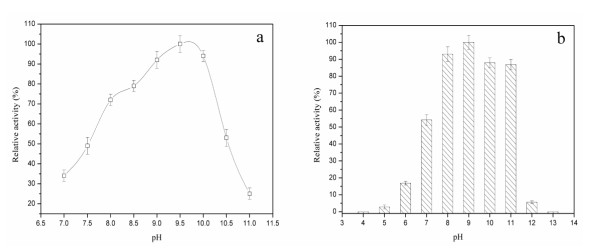
**Effect of pH on the activity and stability of alkaline α-amylase**. a: Effect of pH on the activity of alkaline α-amylase. The purified alkaline α-amylase was incubated and determined in 0.1 M glycine/NaOH buffer (pH 7.0-11.0) at 55°C. b: Effect of pH on the stability of alkaline α-amylase. The pH stability of alkaline α-amylase was determined at pH ranging from 4.0 to 13.0 (0.1 M glycine/HCl buffer or glycine/NaOH buffer) at 25°C for 24 h. After incubation, the activity of alkaline α-amylase was measured at pH 10.0 and 55°C.

Effect of pH on the activity and stability of recombinant alkaline α-amylase was determined. As shown in Figure 2(a), the optimum pH of recombinant alkaline α-amylase was around 9.8. The activity of alkaline α-amylase increased with the increase of pH from 7.0 to 9.8, and exhibited the highest value (> 90% of maximum) between pH 9.0 and 10.0. The activity of alkaline α-amylase rapidly declined when pH was lower than 7.0 or higher than 10.0.

Figure 2(b) shows that the recombinant alkaline α-amylase was very stable, retaining more than 85% of its maximal activity between pH 8.0 and 11.0 after incubation at 25°C for 24 h, and displayed optimal stability at around pH 8.6. Less than 20% of its maximal activity was retained when pH was lower than 7.0 or higher than 11.0, and the activity was completely lost when the pH was at 4.0 or 13.0.

**Figure 3:**
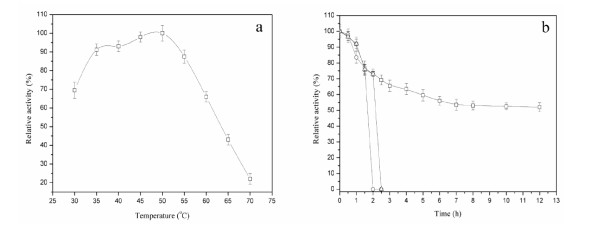
**Effect of temperature on the activity and stability of alkaline α-amylase**. a: Effect of temperature on the activity of alkaline α-amylase. To determine the optimal temperature of alkaline α-amylase, the reaction was conducted from 30 to 70°C in glycine/NaOH buffer (pH 10.0). b: Effect of temperature on the stability of alkaline α-amylase. Square: 30°C; Triangle: 40°C; Circle: 50°C. The thermal stability of alkaline α-amylase was determined at the indicated temperatures in glycine/NaOH buffer (pH 10.0) for 12 h. After incubation, the activity of alkaline α-amylase was measured at pH 10.0 and 55°C.

Figure 3(a) shows that the recombinant alkaline α-amylase activity increased with temperature increasing from 30 to 50°C and decreased with temperature increasing from 50 to 70°C. The optimum temperature was 50°C, at which the activity of alkaline α-amylase was 4.6 times that at 70°C. The alkaline α-amylase retained approximately 60% of activity after incubation at 30°C at pH 9.0 for 12 h or at 40°C at pH 9.0 for 2 h, but was rapidly inactivated above 50°C (Figure 3(b)). The half time of alkaline α-amylase was more than 12 h at 30°C, whereas decreased to 2 and 1.5 h when at 40 and 50°C, respectively.

### The kinetic parameters of recombinant alkaline α-amylase

**Figure 4:**
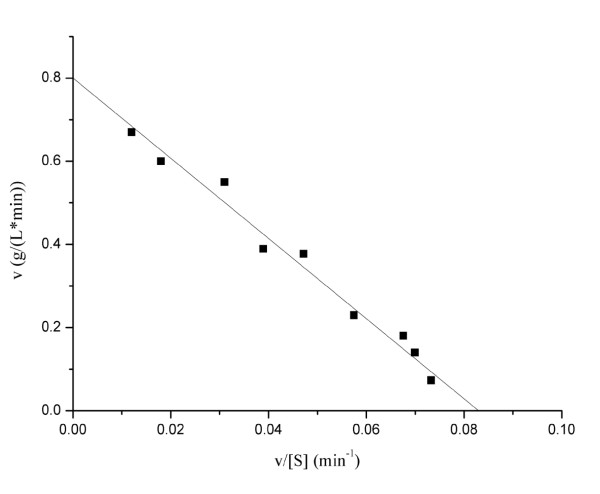
**Eadie-Hofstee plots for soluble starch degradation by alkaline α-amylase**. The soluble starch was used as the substrate with different concentrations ranging from 1 to 10 g/L. The determination of kinetic parameters was performed in glycine/NaOH buffer (pH 10.0) at 55°C.

The kinetics of the recombinant alkaline α-amylase was analyzed using soluble starch as substrate. The *K*_m _and *V*_max _valves were determined by nonlinear fit analysis based on Eadie-Hofstee plots [30]. The initial reaction rates were determined with soluble starch as the substrate with the concentration ranging from 1.0 to 10.0 g/L. The recombinant alkaline α-amylase hydrolyzed soluble starch with Michaelis constant (*K*_m_) of 9.64 g/L and maximum reaction rate (*V*_max_) of 0.80 g/(L·min) at 55°C (Figure 4). The turnover number (*k*_cat_) and the catalytic efficiency (*k*_cat _/*K*_m_) of the alkaline α-amylase were 7.53 min^-1 ^and 0.78 L/(g·min), respectively.

### Effect of metal ions on the stability of alkaline α-amylase

Metal ions played a key role in protein folding and catalysis [31]. As shown in Table 1, the purified recombinant alkaline α-amylase was activated by 1 mM Na^+^, indicating that the alkaline α-amylase activity was Na^+ ^-dependent with a relative activity of 110%. The Ca^2+ ^(1 mM) and K^+ ^(10 mM) almost had no activation or inhibition on the alkaline α-amylase with a relative activity of 102% and 100%, respectively. The following metal ions had slight inhibitions on enzyme activity: Na^+ ^(10 mM), Ca^2+ ^(10 mM), Co^2+ ^(1 and 10 mM), Zn^2+ ^(1 and 10 mM), Mg^2+ ^(1 and 10 mM), Cu^2+ ^(1 and 10 mM), Fe^3+ ^(1 and 10 mM), K^+ ^(1 mM) and Fe^2+ ^(10 mM). On the other hand, the stronger inhibitory effects were observed in the presence of Fe^2+ ^(1 mM) and Mn^2+ ^(1 and 10 mM), and only 26.5% of activity was retained in the presence of 10 mM Mn^2+^. The inhibitions of divalent metal ions on α-amylase from the other sources were also observed [32-35].

**Table 1:** Effect of metal ions on the stability of alkaline α-amylase

Metal ions	**Relative activity (%)**^**b**^	
	
	1 mM	10 mM
CK^a^	100	100
Na^+^	110 ± 2	96 ± 2
Co^2+^	97 ± 2	96 ± 2
Zn^2+^	80 ± 1	85 ± 1
Ca^2+^	102 ± 2	85 ± 3
K^+^	97 ± 2	100 ± 2
Mn^2+^	37 ± 1	27 ± 3
Mg^2+^	86 ± 2	84 ± 2
Fe^2+^	74 ± 2	84 ± 2
Cu^2+^	81 ± 1	88 ± 1
Fe^3+^	90 ± 2	86 ± 1

^a ^The relative activity was calculated on the basis of the activity (CK) obtained in glycine/NaOH buffer (pH 10.0) without the addition of any metal ions.

^b ^Values represent means ± SD (n = 3) relative to the untreated control samples.

### Culture conditions optimization for the overproduction of alkaline α-amylase by *B. subtilis*

**Figure 5:**
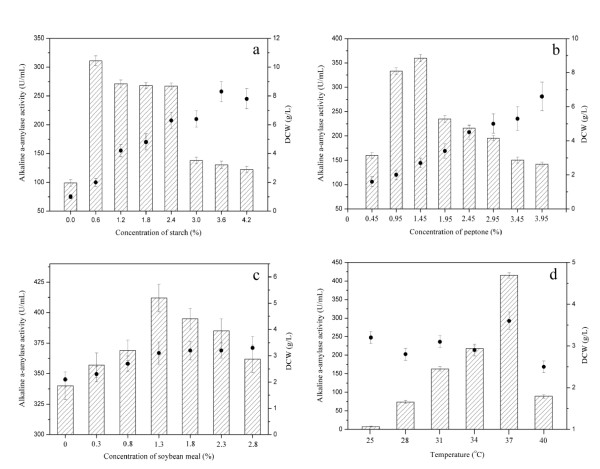
**Optimization of medium constituents and process conditions for alkaline α-amylase production by *B. subtilis***. Histogram: alkaline α-amylase activity; Black dot: dry cell weight (DCW). a: Effect of starch on the recombinant α-alkaline amylase production by *B. subtilis*. Different concentrations of starch added into the medium were 0, 0.6, 1.2, 1.8, 2.4, 3.0, 3.6 and 4.2% (w/v). b: Effect of peptone concentrations on the recombinant production of α-alkaline amylase by *B. subtilis*. The different concentrations of peptone were 0.45, 0.95, 1.45, 1.95, 2.45, 2.95, 3.45 and 3.95% (w/v). c: Effect of soybean meal concentrations on the α-alkaline amylase production by *B. subtilis*. The different concentrations of soybean meal were 0, 0.3, 0.8, 1.3, 1.8, 2.3 and 2.8% (w/v). d: Effect of temperature on the recombinant α-alkaline amylase production by *B. subtilis*. The different temperatures were 25, 28, 31, 34, 37 and 40°C.

Figure 5(a) shows the influence of starch concentrations (0, 0.6, 1.2, 1.8, 2.4, 3.0, 3.6, and 4.2% (w/v)) on the yield of recombinant alkaline α-amylase. The yield of alkaline α-amylase increased with the increase of starch concentration in a range from 0.0 to 0.6%, and the maximal alkaline α-amylase yield reached 311 U/mL at 0.6% starch. Alkaline α-amylase yield decreased when more than 0.6% starch was added. The dry cell weight (DCW) increased with the increase of starch concentration up to 3.6%.

As shown in Figure 5(b), the peptone of different concentrations (0.45, 0.95, 1.45, 1.95, 2.45, 2.95, 3.45, and 3.95% (w/v)) was added into the medium. The addition of peptone within a suitable concentration range can greatly enhance the yield of alkaline α-amylase, and the maximal alkaline α-amylase production reached 360 U/mL when 1.45% peptone was added. The cell density increased with the increase of peptone concentration. However, alkaline α-amylase yield decreased when the peptone concentration was higher than 1.45%.

The recombinant alkaline α-amylase activity increased with the increase of soybean meal concentration, and reached the highest level of 412 U/mL at 1.3% soybean meal (Figure 5(c)). The further increase of soybean meal concentration higher than 1.3% was not favorable for alkaline α-amylase production. The cell density increased with the increase of soybean meal concentration, and reached the highest level of 3.3 g/L at 2.8% soybean meal.

As shown in Figure 5(d), the effects of temperature (25, 28, 31, 34, 37, and 40°C) on cell growth and alkaline α-amylase production were investigated. The highest alkaline α-amylase yield (415 U/mL) was observed at 37°C, meanwhile the maximal cell density (3.6 g/L) of *B. subtilis *also reached at 37°C. The yield of recombinant alkaline α-amylase significantly decreased at lower temperatures (below 37°C) or higher temperatures (above 37°C). The temperature was very important for the recombinant alkaline α-amylase expression in *B. subtilis*.

### Production of alkaline α-amylase by recombinant *B. subtilis *in a 3-L fermentor

**Figure 6:**
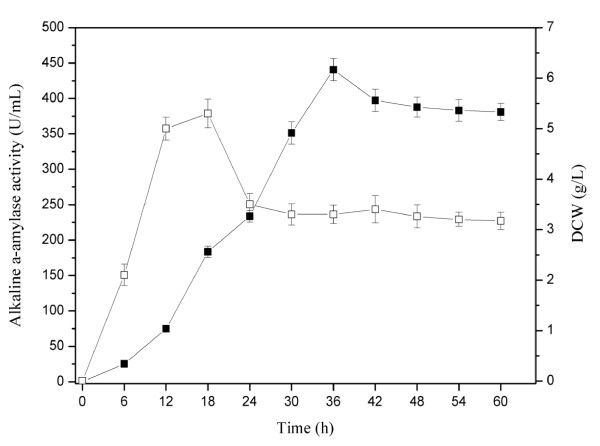
**Time profiles for batch cultivation of recombinant *B. subtilis *in 3 L fermentor**. Black square: the activity of alkaline α-amylase (U/mL); White square: DCW (g/L).

The expression efficiency of the engineered *B. subtilis *was further explored in a 3-L fermentor (BIOTRON, Korea). As shown in Figure 6, during the growth phase, the maximum cell concentrations in the fermentor reached 5.3 g/L. The expression of alkaline α-amylase was continuously increased and reached the maximum yield of 441 U/mL at 36 h. This yield was 79 times that of native alkaline α-amylase extracted from *Bacillus alcalophilus *JN21 and 8.5 times that of alkaline α-amylase from recombinant *E. coli *[7]. The high titer of alkaline α-amylase indicated that *B. subtilis *was a suitable host for the industrial production of alkaline α-amylase.

## Conclusions

In this work, the gene of alkaline α-amylase from *B. alcalophilus *JN21 was cloned and expressed in *B. subtilis *WB600. The optimum pH of recombinant alkaline α-amylase was 9.5, and it displayed optimal stability at pH 9.0. The optimal temperature of the alkaline α-amylase was 50°C, and it was stable below 40°C. The recombinant alkaline α-amylase hydrolyzed soluble starch with *K*_m _of 9.64 g/L and *V*_max _of 0.80 g/(L·min). The alkaline α-amylase was slightly activated by 1 mM Na^+^, 1 mM Ca^2+ ^or 10 mM K^+ ^but inhibited by other metal ions. Under the optimal conditions (starch 0.6%, peptone 1.45%, soybean meal 1.3%, and temperature 37°C), the highest yield of alkaline α-amylase reached 415 U/mL, which was 4.2-fold that before optimization. The yield of alkaline α-amylase in a 3-L fermentor reached 441 U/mL.

## Materials and methods

### Plasmids, bacterial strains and materials

The strain of *Bacillus alcalophilus *JN21 (CCTCC NO: M 2011229) was an isolate of Lab of Bioprocess and Biosystems Engineering, Jiangnan University. *Bacillus subtilis *WB600, a strain that contains deletions of all six extracellular protease genes, and the plasmid pMA5 were obtained from Invitrogen. The EZ-10Spin Column Plasmid Mini-Preps kit, agarose gel DNA purification kit, restriction enzymes, and T4 DNA ligase were obtained from TakaRa (Dalian, China). Other chemicals were obtained from Shanghai Sangon Biological Engineering Technology & Services Co. Ltd. (Shanghai, China). The synthesis of DNA primers and DNA sequencing were performed by Shanghai Sangon Biological Engineering Technology & Services Co. Ltd. (Shanghai, China).

### Construction and transformation of the plasmid for secreted alkaline α-amylase expression

The *E. coli/B. subtilis *shuttle vector (pMA5) was used to clone and express alkaline α-amylase. The mature alkaline α-amylase gene was amplified by polymerase chain reaction (PCR) using gene-specific primers as follows: 5'ACGCGTCGACATGGAGCATASGGCCATGACGA3' (forward primer) and 5'CGGGATCCTTATGACCGCCGAATCAGTGAAGC3' (reverse primer). The forward primer and the reverse primer contained a *Sal *I and a *Bam*H I restriction sites (underlined), respectively. The amplification was carried out under the following conditions: the first step was at 94°C for 10 min, followed by 30 cycles of 95°C for 10 s, 60°C for 30 s, and 72°C for 2 min, and the final extension was carried out at 72°C for 10 min. The PCR product was digested with *Sal *I and *Bam*H I, gel-purified and then ligated into pMA5 which was subjected to a similar treation. The recombinant plasmid was identified by restriction analysis and sequencing.

### Expression of alkaline α-amylase in *B. subtilis*

The recombinant plasmid pMA5/*A-amyQ *was transformed into the competent cells of *Bacillus subtilis *WB600. The transformants were selected at 37°C on the LB agar plates containing 100 μg/mL Kanamycin and 1% soluble starch for 8 h. The transformants with transparent rings around colonies were positive. The presence of alkaline α-amylase gene in the transformants was confirmed by PCR. The colonies were grown in 25 mL of LB medium at 37°C for 36 h in conical flasks (250 mL).

### Optimization of recombinant alkaline α-amylase production

The effects of culture conditions (starch concentration, peptone concentration, soybean meal concentration, and temperature) on the recombinant production of alkaline α-amylase in *B. subtilis *were examined. The starch of different concentrations (0, 0.6, 1.2, 1.8, 2.4, 3.0, 3.6, and 4.2% (w/v)) on the production of alkaline α-amylase was added into the culture medium to examine the effects of starch on alkaline α-amylase production. The peptone of different concentrations (0.45, 0.95, 1.45, 1.95, 2.45, 2.95, 3.45, and 3.95% (w/v)) was also added into the culture medium to examine the effects of peptone on alkaline α-amylase production. Soybean meal of different concentrations (0, 0.3, 0.8, 1.3, 1.8, 2.3, and 2.8% (w/v)) was added into the culture medium to investigate the effects of soybean meal on the alkaline α-amylase production. The effects of temperature (25, 28, 31, 34, 37, and 40°C) on the production of alkaline α-amylase were also investigated.

### Production of alkaline α-amylase by recombinant *B. subtilis *in a 3-L fermentor

The recombinant *B. subtilis *with the highest alkaline α-amylase yield was used to scale up fermentation in a 3 L fermentor (BIOTRON, Korea). The medium was supplemented with 1.45% peptone, 0.6% soluble starch, 1.3% soybean meal, 1.0% NaCl and 100 μg/mL Kanamycin. The fermentation was performed with a working volume of 1.6 L at 37°C for 60 h. The stirring speed of fermentation was 600 rpm, and the volume of ventilation was 1 vvm.

### Enzyme assays

Alkaline α-amylase was determined by measuring the amount of reducing sugar released during enzymatic hydrolysis of 1% soluble starch in glycine/NaOH buffer (pH 10.0) at 55°C for 5 min. A control without enzyme addition was used. The amount of reducing sugar was measured by a modified dinitrosalicylic acid method [36]. One unit of alkaline α-amylase activity was defined as the amount of enzyme that released 1 μmol of reducing sugar as glucose per min at the assay conditions.

### Purification and molecular weight determination of alkaline α-amylase

Solid ammonium sulfate was added to the supernatant to 70% saturation at 0°C. The precipitate was collected and dissolved in glycine/NaOH buffer (pH 9.0) and dialyzed overnight against the same buffer. After dialysis, the enzyme solution was filtered using water film. And then the enzyme solution was injected into AKTA purifier (GE Healthcare USA) through the anion exchange (Q-Sepharose HP). The subunit molecular weight of recombinant alkaline α-amylase was determined by SDS-PAGE.

### Determination of kinetic parameters

The reaction was performed in glycine/NaOH buffer (pH 10.0) at 55°C for the determination of kinetic parameters. Assays were performed with active enzyme and soluble starch of different concentrations from 1 to 10 g/L. The Eadie-Hofstee plots were used to calculate kinetic parameters *K*_m _and *V*_max _according to the enzyme reactions [30].

### Effects of temperature and pH on activity

To determine the optimal temperature, the enzyme was analyzed from 30 to 90°C in glycine/NaOH buffer (pH 10.0). The thermal stability of alkaline α-amylase was determined at the indicated temperatures in glycine/NaOH buffer (pH 10.0). To estimate the optimal pH for the alkaline α-amylase, the purified protein was incubated in various buffers. The buffer used for determination of optimal pH was as follows: glycine/HCl buffer for pH 3.0-6.0, glycine/NaOH buffer for pH 6.0-11.0. The pH stability of alkaline α-amylase was determined at pH ranging from 3.0 to 11.0 at 25°C for 24 h. After incubation, the alkaline α-amylase activity was measured at pH 10.0 and 55°C.

### Effects of metal ions on enzyme activity

Alkaline α-amylase was pre-incubated with 1 mM and 10 mM KCl, NaCl, CaCl_2_, MgCl_2_, FeCl_3_, FeCl_2_, CoCl_2_, ZnCl_2_, MnCl_2 _and CuCl_2_, respectively. The relative activity was determined and compared with the activity obtained in glycine/NaOH buffer (pH 10.0) without the addition of any metal ions.

### Protein content assay

The protein content of samples was measured by the Bradford method with BSA as a standard [37].

## Competing interests

The authors declare that they have no competing interests.

## Authors' contributions

HQY carried out the cloning and expression of alkaline α-amylase, and drafted the manuscript. JHL carried out the fermentation. LL participated in the statistical analysis of data. GCD and JC critically revised and corrected the manuscript. All authors read and approved the final manuscript.
